# Risk factors for the transmission of foot-and-mouth disease during the 2010 outbreak in Japan: a case–control study

**DOI:** 10.1186/1746-6148-9-150

**Published:** 2013-07-24

**Authors:** Norihiko Muroga, Sota Kobayashi, Takeshi Nishida, Yoko Hayama, Takaaki Kawano, Takehisa Yamamoto, Toshiyuki Tsutsui

**Affiliations:** 1 Viral Disease and Epidemiology Research Division, National Institute of Animal Health, National Agriculture and Food Research Organization, 3-1-5 Kannondai, Tsukuba, Ibaraki 305-0856, Japan; 2 Animal Products Safety Division, Food Safety and Consumer Affairs Bureau Ministry of Agriculture, Forestry and Fisheries, 1-2-1 Kasumigaseki, Chiyoda-ku, Tokyo 100-8950, Japan; 3 Miyazaki Livestock Hygiene Service Center, 3151-1 Shimonaka, Sadowara-cho, Miyazaki, Miyazaki 880-0212, Japan

**Keywords:** Case–control study, Foot-and-mouth disease, Transmission, Risk factors, Japan

## Abstract

**Background:**

In 2010, foot-and-mouth disease (FMD) occurred for the first time in a decade in Japan. Movement or shipment of people and animals around infected farms was restricted; however these contingency measures proved insufficient to prevent FMD spread. Consequently, a total of 292 farms were confirmed as infected during this outbreak. We conducted a case–control study to identify the risk factors associated with FMD transmission between farms during these restrictions. As there was discordance in the control measures taken, risk factors were examined separately for two areas. Analyses were also performed separately for cattle and pig farms given their different infectivity and susceptibility.

**Results:**

For cattle farms in the movement restriction area, the odds of having the factor ‘farm equipment was shared with other farms’ was significantly higher for case farms than for control farms. For cattle farms in the shipment restriction area, the odds of having the factors ‘feed transport vehicles visited the farm’ and ‘staff of livestock-related companies visited the farm’ were significantly higher on case farms than control farms. In pig farms in the movement restriction area, the odds of having factor ‘farm staff commuted from outside’ was 20 times higher for case farms than control farms. In addition, case farms were less likely to have the factors ‘fattening farm’ and ‘barn has physical barriers’ compared with control farms.

**Conclusions:**

In the movement restriction area, the disease was likely to spread regardless of the movement of people and vehicles, and physical barriers were found to be a protective factor. Therefore, physical barriers from the surrounding environments seemed to prevent farms from being infected. Conversely, in the shipment restriction area, movement of people and vehicles was strongly associated with disease spread. These results allow a better understanding of the risk factors associated with FMD transmission and are useful to enhance future preventive measures against transmission during FMD outbreaks.

## Background

Foot-and-mouth disease (FMD) is a highly contagious viral disease affecting cloven-hoofed animals. Characteristic clinical signs of FMD include pyrexia, salivation, and lameness, with vesicles and erosions in the mouth, on the feet, and on the teats [1-3]. Once FMD invades a FMD-free country, susceptible animals on infected farms and surrounding areas are often culled to eradicate the disease. This causes serious economic losses to farmers and livestock industries, both from direct losses and the suspension of international trade in animal products.

**Figure 1:**
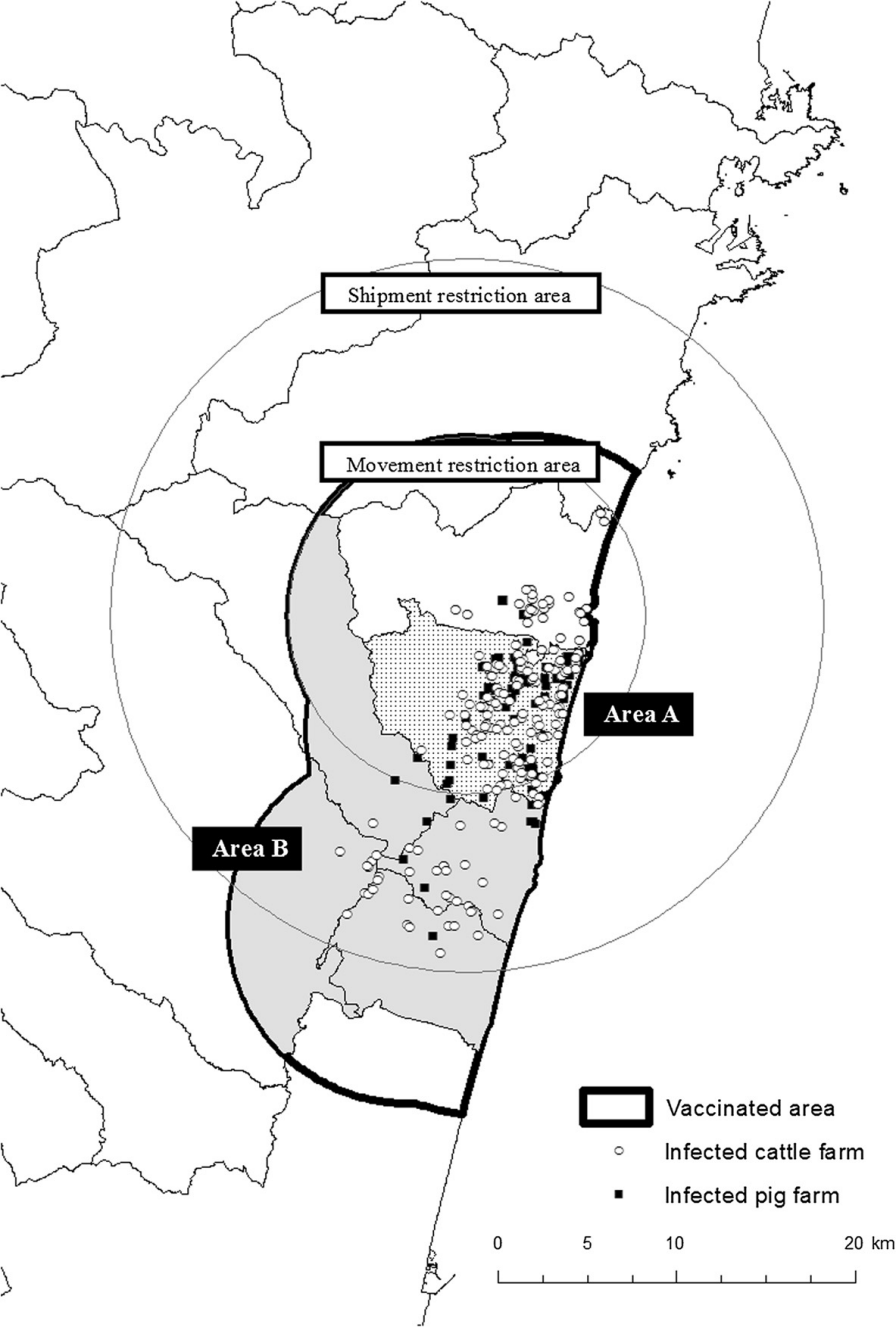
**Major epidemic area during the 2010 FMD outbreak in Japan.** Infected farms, vaccinated areas, and movement and shipment restriction areas are depicted at the point of the first detection of the disease.

In April 2010, there was a large-scale FMD outbreak in Miyazaki Prefecture in Japan. Miyazaki Prefecture was the primary livestock production area of the country [4], with 315,000 cattle at a density of 165.0 farms/100 km^2^ and 915,000 pigs at a density of 9.8 farms/100 km^2^. A total of 292 infected farms were detected before the last case on July 4, 2010, and nearly 300,000 animals, including vaccinated animals, were culled. The major epidemic area extended 20 km from north to south in Miyazaki Prefecture (Figure 1). The index case was detected in the town of Tsuno in the northern part of the area on April 20. Subsequent cases were detected on cattle farms in the town of Kawaminami, adjacent to Tsuno. This area had one of the highest densities of cattle and pig farms in the region [5] and became the most affected area, with 197 confirmed infected farms (126 cattle farms, 70 pig farms, and 1 goat farm).

On the 24th day of the epidemic, the disease spread to the neighboring towns south of Kawaminami and infection was confirmed on 55 farms (45 cattle farms and 10 pig farms) in this southern area. Emergency vaccination was implemented between May 22 and 26 for all cattle and pig farms in a 10-km radius from the infected farms. These vaccinated animals were subsequently culled to restrict further spread of the disease. After vaccination, the number of detected farms per day decreased and the epidemic ended.

During this epidemic, stamping out and movement restrictions were implemented as containment measures. As soon as a farm was diagnosed as infected, all animals on the farm were culled and a surrounding 10-km radius movement restriction area was established. Within the movement restriction area, movement of all cloven-hoofed animals, carcasses, feces, farm equipment, and other commodities that could transmit FMD virus was prohibited. In addition, a shipment restriction area was established in a 10–20-km-wide ring surrounding the movement restriction area. In this area, movement of cloven-hoofed animals, vehicles, people or other equipment inside the area was allowed, although movement of animals and commodities out of the restriction area was prohibited.

As the disease continued to spread despite implementation of restrictions in the 2010 epidemic, understanding the risk factors associated with FMD transmission after the implementation of restrictions is necessary to determine how to prevent this in future outbreaks. We conducted a case–control study to elucidate these factors dividing the study area into two geographic regions according to the different control measures in place and species of animals on farms.

## Methods

### Study design

#### Case and control farm definition

A case control study was designed to examine risk factors associated with FMD transmission between farms within the major epidemic area (vaccinated area; 280 infected farms). We focused on the time period between the implementation of the movement restrictions and the start of emergency vaccination as vaccinated animals may have different susceptibility to the infection compared with naive animals. Therefore, 19 farms assumed to be infected before the implementation of movement restrictions and 6 farms where the date of infection was unclear were excluded from this study. Animals suspected of FMD infection were diagnosed by reverse transcription- polymerase chain reaction (RT-PCR). ‘Case farms’ were selected from positive farms, which had the animals diagnosed as infected with FMD. ‘Control farms’ were selected from the negative farms, which had no animals diagnosed as infected with FMD until the end of the epidemic.

### Study population

In the epidemic area, control measures were largely different between Kawaminami and the four towns to the south – Takanabe, Kijo, Shintomi, and Saito. Therefore, we divided the study population into two geographic areas: Kawaminami, which was in the movement restriction area (area A), and the four southern towns, which were in the shipment restriction area (area B) (Figure 1).

In addition, because of differences in infectivity and susceptibility between cattle and pigs [2,3] and differences in farming practices between cattle and pig farms, the risk factors associated with FMD infection were examined independently between cattle farms and pig farms. This study specifically targeted beef farms because the number of dairy farms was small (6.7% of the total cattle farms). In area B, the study population was limited to cattle farms because there were few pig farms (6.3% of the total farms). As a result, the study population was categorized into three groups: cattle farms in area A, cattle farms in area B, and pig farms in area A. The target farms were randomly selected in each group. Given the limitations of resources available for farm investigations, we consequently employed a total of 281 farms. In cattle farms from area A, 49 out of 100 positive farms and 49 out of 105 negative farms were included in the study as cases and controls, respectively. One negative farm declined to participate, yielding 49 case and 48 control farms. In cattle farms from area B, all 37 positive farms were included as cases. For controls, 74 farms were randomly selected from 426 negative farms; however, one farm declined to participate, giving 73 farms as controls. In pig farms from area A, there were 62 farms and 17 negative farms, all of which were included in the study. The information for 3 positive farms was unavailable and 2 negative farms declined to participate, resulting in a total of 59 case and 15 control farms.

### Data collection

Farm investigations were conducted using a questionnaire composed of 5 sections with 20 risk factors associated with transmission between farms: 1) general farm information, 2) movement of people, 3) movement of vehicles, 4) farm management, and 5) farm location with the consent of famers about this study (Table 1) [Additional file 1]. This questionnaire was constructed based on the potential risk factors reported in past studies [6-8].

**Table 1:** Brief description of the questionnaire used in the case control study

**Parameters**	**Description**	**Responses**
General farm information	Herd size	large or small
	Fattening farm (only pig farms)	yes or no
	Belong to a company group (only pig farms)	yes or no
People movement	Farmer visited other livestock farms	yes or no
	Farm staff commuted from outside	yes or no
	Veterinarians visited the farm	yes or no
	Agricultural technicians visited the farm	yes or no
	Staff of livestock related companies (such as drug companies) visited the farm	yes or no
	Other livestock farmers visited the farm	yes or no
	Other people (such as relative and town hall staff) visited the farm	yes or no
Vehicle movement	Feed transport vehicles visited the farm	yes or no
	Farmer transported feed by own vehicle	yes or no
	Carcass transport vehicles visited the farm	yes or no
	Bedding transport vehicles visited the farm	yes or no
Farm management	Farm equipment (such as tractors and roll balers) were shared with other farms	yes or no
	Manure was removed from the farm	yes or no
Farm location	Barn has physical barriers (such as a private house and clump of trees)	yes or no
	A forest surrounds the farm	yes or no
	Housing is located outside the farm	yes or no
	Barns bordered by a road	yes or no

Data from control farms were collected by visiting each farm and interviewing the farm manager in May and June 2011. The survey covered events between April 20, 2010 and July 27, 2010, when movement restrictions were implemented. Data from case farms were collected during the on-site investigation by visiting farms and interviewing farm managers during the epidemic. Missing information was obtained via subsequent telephone interviews. On-site investigators evaluated questions about farm environment, such as ‘a forest surrounds the farm and barn has physical barriers’.

### Statistical analyses

A continuous variable, the herd size, was categorized into two ranks using the median value for each group (24, 24, and 814 animals for cattle farms in area A, cattle farms in area B, and pig farms in area A, respectively). All binary and categorical explanatory variables were examined by univariable analysis using a chi-squared or Fisher’s exact test.

Multivariable analysis was conducted using a logistic regression model. To select the explanatory variables for the multivariable analysis, the method described by Dohoo et al. [9] was applied. Variables with p-values < 0.15 in a univariable analysis were selected as candidates for the multivariable analysis. Correlations between candidate variables for multivariable analysis were examined using the phi coefficient. Of variables that were highly correlated (phi coefficient > 0.4), the more reasonable variable was selected for the multivariable analysis. Herd size was forced into all logistic regression models because it was considered to be a potential confounding factor.

In the logistic regression model, variables were selected using a stepwise backward elimination approach, using p < 0.05 for retention, and interactions between variables in the final model were assessed. The final model was checked for goodness-of-fit using Hosmer–Lemeshow statistics [10]. All statistical analyses were conducted using SPSS version 20.0 (SPSS Inc., Chicago, IL, USA).

This study protocol was reviewed and approved by the Ministry of Agriculture, Forestry and Fisheries of Japan and Miyazaki prefecture.

## Results

### Cattle farms in area A

The results of univariable analyses on cattle farms in area A are shown in Table 2. Large farms represented 31 out of 49 case farms, but only 18 of 48 control farms. People movements were rarely observed in either case or control farms. Among variables regarding farm management and farm locations, ‘farm equipment was shared with other farms’ and ‘a forest surrounds the farm’ were significantly associated with FMD infected farms.

**Table 2:** Results of univariable analyses for risk factors associated with FMD transmission between farms

	**Level**	**Cattle**						**Pigs**		
		**Area A**			**Area B**			**Area A**		
		**Number of farms**		**P-value**	**Number of farms**		**P-value**	**Number of farms**		**P-value**
		**Case**	**Control**		**Case**	**Control**		**Case**	**Control**	
Total		49	48		37	73		59	15	
General information on the farm										
Herd size	large	31	18	0.011	35	21	<0.001	31	6	0.386
	small	18	30		2	52		28	9	
Fattening farm	yes	-	-	-	-	-	-	13	9	0.007^*^
	no	-	-		-	-		46	6	
Belong to a company group	yes	-	-	-	-	-	-	8	7	0.009
	no	-	-		-	-		51	8	
People movements										
Farmer visited other livestock farms	yes	4	4	0.631	4	12	0.429	9	3	0.457
	no	45	44		33	61		50	12	
Farm staff commuted from outside	yes	0	0	-	16	1	<0.001^*^	22	1	0.018^*^
	no	49	48		21	72		37	14	
Veterinarians visited the farm	yes	3	4	0.488	10	5	0.004	0	1	0.203
	no	46	44		27	68		59	14	
Agricultural technicians visited the farm	yes	0	4	0.056^*^	3	4	0.437	0	0	-
	no	49	44		34	69		59	15	
Staff of livestock related companies visited the farm	yes	0	3	0.117^*^	13	1	<0.001^*^	18	3	0.323
	no	49	45		24	72		41	12	
Other livestock farmers visited the farm	yes	1	4	0.175	1	6	0.249	0	1	0.203
	no	48	44		36	67		59	14	
Other people visited the farm	yes	6	2	0.141^*^	5	6	0.289	8	0	0.147^*^
	no	43	46		32	67		51	15	
Vehicle movements										
Feed transport vehicles visited the farm	yes	7	4	0.355	26	14	<0.001^*^	33	8	0.857
	no	42	44		11	59		26	7	
Farmer transported feed with own vehicle	yes	26	22	0.477	3	22	0.009^*^	9	1	0.35
	no	23	26		34	51		50	14	
Carcass transport vehicles visited the farm	yes	1	0	0.505	12	2	<0.001^*^	3	1	0.604
	no	48	48		25	71		56	14	
Bedding transport vehicles visited the farm	yes	15	14	0.876	23	19	<0.001^*^	24	5	0.603
	no	34	34		14	54		35	10	
Farm management										
Farm equipment was shared with other farms	yes	10	1	0.004^*^	4	2	0.097^*^	1	1	0.367
	no	39	47		33	71		58	14	
Manure was removed from the farm	yes	7	10	0.396	3	11	0.237	9	1	0.35
	no	42	38		34	62		50	14	
Farm location										
Barn has physical barriers	yes	21	30	0.053^*^	26	49	0.738	30	12	0.042^*^
	no	28	18		11	24		29	3	
A forest surrounds the farm	yes	31	40	0.026^*^	25	57	0.232	34	12	0.111^*^
	no	18	8		12	16		25	3	
Housing located outside the farm	yes	5	2	0.226	19	10	<0.001^*^	23	5	0.687
	no	44	46		18	63		36	10	
Barns bordered by a road	yes	17	21	0.361	18	18	0.011^*^	33	4	0.043^*^
	no	32	27		19	55		26	11	

* Variables included into a multivariable logistic regression model.

A total of six explanatory variables (p < 0.15) were selected for the multivariable analysis: ‘farm equipment was shared with other farms’, ‘other people visited the farm’, ‘staff of livestock related companies visited the farm’, and ‘agricultural technicians visited the farm’ as risk factors and ‘barn has physical barriers’ and ‘a forest surrounds the farm’ as protective factors. The odds of case farms having the factor ‘farm equipment was shared with other farms’ was significantly higher than control farms (OR: 9.6, 95%CI: 1.1–80.2) (Table 3).

**Table 3:** Results of multivariable analysis for FMD transmission between farms

**Group**	**Variables**	**Level**	**Odds ratio**	**95% Confidence interval**	**P-value**
Cattle					
Area A^1)^	herd size	large	2.8	1.2-6.7	0.02
		small	1.0		
	farm equipment was shared with other farms	Yes	9.6	1.1-80.2	0.04
		No	1.0		
	Constant			-	0.54
Area B^2)^	herd size	large	28.8	5.8-143.4	<0.01
		small	1.0		
	staff of livestock related companies visited the farm	Yes	20.4	1.1-383.0	0.04
		No	1.0		
	feed transport vehicles visited the farm	Yes	5.1	1.5-16.7	0.01
		No	1.0		
	Constant			-	<0.01
Pig					
Area A^3)^	herd size	large	0.7	0.2-3.3	0.70
		small	1.0		
	fattening farm	Yes	0.1	0.0-0.4	<0.01
		No	1.0		
	farm staff commuted from outside	Yes	20.0	1.8-226.9	0.02
		No	1.0		
	barn has physical barriers	Yes	0.1	0.0-0.5	<0.01
		No	1.0		
	Constant			-	0.15

1) Hosmer-Lemeshow goodness-of-fit chi-square score = 0.1(d.f. = 2, p = 0.97).

2) Hosmer-Lemeshow goodness-of-fit chi-square score = 3.6 (d.f. = 3, p = 0.30).

3) Hosmer-Lemeshow goodness-of-fit chi-square score = 5.6 (d.f. = 7, p = 0.59).

### Cattle farms in area B

Table 2 shows the results of univariable analyses in cattle farms in area B. Of the 10 variables with p < 0.15 in the univariable analysis, ‘veterinarians visited the farm’ was not selected for the multivariable analysis because this variable was highly correlated with ‘carcass transport vehicles visited the farm’ (phi coefficient = 0.59). We reasoned that a veterinarian always attended when carcasses were transported, and ‘carcass transport vehicles visited the farm’ was considered more likely to be a risk factor. Therefore, nine explanatory variables were selected for the multivariable analysis; ‘farm staff commuted from outside’, ‘staff of livestock related companies visited the farm’, ‘feed transport vehicles visited the farm’, ‘farmer transported feed by own vehicle’, ‘carcass transport vehicles visited the farm’, ‘bedding transport vehicles visited the farm’, ‘farm equipment was shared with other farms’, ‘housing is located outside of the farm’, and ‘barns bordered by a road’. Then, as a result of the multivariable analysis, the odds of case farms having the factor ‘feed transport vehicles visited the farm’ was OR = 5.1 (95%CI: 1.5–16.7, p = 0.01) and ‘staff of livestock related companies visited the farm’ was OR = 20.4 (95%CI: 1.1–383.0, p = 0.04) compared with control farms (Table 3).

### Pig farms in area A

For pig farms in area A, ‘farm staff commuted from outside’ and ‘fattening farm’ were associated with transmission of FMD between farms. Among farm location variables, ‘barn has physical barriers’ and ‘barns bordered by a road’ were associated with transmission.

Among the seven variables with p-values < 0.15 in the univariable analysis, ‘fattening farm and ‘belonging to a company group’ were highly correlated (phi coefficient = 0.49) because farms belonging to a company group are generally fattening farms. ‘Fattening farm’ was considered to describe a type of farming practice; therefore ‘belonging to a company group’ was dropped from the analysis. Finally, six explanatory variables were selected for the multivariable analysis; ‘farm staff commuted from outside’, ‘barns bordered by a road’, and ‘other people visited the farm’ as risk factors and ‘fattening farm’, ‘barn has physical barriers’, and ‘a forest surrounds the farm’ as protective factors. The results of the final model showed that the odds of case farms having the factor ‘farm staff commuted from outside’ was significantly higher than control farms, with OR = 20.0 (95%CI: 1.8-226.9). The factors ‘fattening farm’ and ‘barn has physical barriers’ were negatively associated with the transmission of disease, with an OR = 0.1 (95%CI: 0.0–0.4) and 0.1 (95%CI: 0.0–0.5), respectively (Table 3).

## Discussion

This case–control study was conducted to investigate risk factors associated with transmission of FMD between farms during the epidemic in Japan in 2010. There were two areas with different control measures: area A, in the movement restriction area, and area B, in the shipment restriction area. We considered the large difference in control measures implemented in those areas would render the transmission mode of FMD incomparable, thus risk factors were separately assessed in these two areas. In area A, physical barriers around farms seemed to be protective factors that reduced FMD transmission. In area B, movements of people and vehicles were indicated as risk factors associated with FMD transmission.

In cattle farms in area B, ‘feed transport vehicles visited the farm’ and ‘staff of livestock related companies visited the farm’ were indicated as risk factors associated with FMD transmission. It is known that movements of people and vehicles are important routes of FMD virus introduction [6,11,12]. Movements found to be associated with FMD transmission were not the type of movement prohibited by the restrictions implemented during the outbreak. Therefore, even in the area of shipment restrictions, FMD transmission could have been effectively controlled by more strict movement restriction and compulsory disinfection of people and vehicles.

However, the movements of people and vehicles were not significant risk factors in area A. This suggests that movements were not associated with transmission, although it may be more reasonable to assume that the disease was spread irrespective of the movement and factors representing the movement were not found to be risk factors. Conversely, ‘barn has physical barriers’ was a protective factor for pig farms in area A. In cattle farms in area A, physical barriers seemed to be protective, although this was statistically marginal. ‘A forest surrounds the farm’ showed a protective effect in univariable analyses. These results suggest that local spread could be the major transmission mode in area A. In the 2001 FMD epidemic in the United Kingdom, local spread played an important role in the dissemination of FMD. Although the exact mechanisms of local spread have not been fully determined, it is believed that the majority of cases result from either local aerosol spread between animals or contamination in an area near an infected farm [7,13]. According to a previous analysis, cattle farms were at a higher risk of becoming infected and pig farms had a higher risk of transmitting virus [14]. The extremely high density of both cattle and pig farms in this area [5] may have facilitated local spread. As the disease might spread regardless of the movement of people and vehicles, implementation of the movement restriction seems to have been insufficient to prevent disease spread in this area. Therefore, control measures against sources of infection, such as immediate destruction of infected farms before an area became highly affected, are essential to prevent the disease spread.

In pig farms in area A, ‘farm staff commuted from outside’ was found to be associated with disease transmission. This factor was also associated with transmission on cattle farms in area B, albeit only in a univariable analysis. Employed farm staff that commuted from their homes located outside of the farm could bring virus to the animals via direct contact.

‘Fattening farm’ was found to be a protective factor for pig farms in area A. Farm workers on fattening farms seemed to enter the livestock barn less frequently than those on breeding or farrow-to-finish farms, probably because sows and piglets in breeding or farrow-to-finish farms require more frequent care than pigs in fattening farms. This would give more chance for FMD virus entry into the barns. These results highlight the critical pathway of disease transmission via direct contact with animals and contaminated fomites. However, these contacts are required for farm operation, therefore particular attention should be paid to ensure thorough disinfection at all entries into barns.

In cattle farms in area A, ‘farm equipment was shared with other farms’ was indicated as a risk factor associated with transmission. This factor was statistically marginal in the univariable analysis for cattle farms in area B. The majority of farm equipment shared in this study were tractors and roll balers that were used in a forage field shared by two or three farms. In these cases, the tractor was brought back to a farm without disinfection and farmers returned to their farms without disinfection of shoes and clothes. As farm equipment movements for purposes other than animal care was not subject to the movement restrictions in the outbreak, this may need to be taken into consideration in future control measures.

We note possible biases due to the case–control study approach based on questionnaires. As risk factors for FMD transmission may have been well known among farmers, it is reasonable to assume this study was prone to recall bias. To minimize this, information obtained from farm managers was supported as much as possible by paper-based records, such as feed or carcass transport vehicle traveling data. This would be also expected to reduce two other biases; the bias arising from the difference in time between the case and control farm investigations and observer bias leading to the misclassification of exposure.

In addition, while case–control studies can identify relationships between possible risk factors and the occurrence of disease, it provides no information about cause and effect. However, for serious infectious diseases like FMD, factors highly correlated with infection should be considered targets of control measures [15,16]. After the 2010 FMD epidemic in Japan, the government strengthened FMD control measures and animal rearing guidelines were altered. Additional control measures included compulsory disinfection of people and vehicles entering rearing areas of the farms and recording visits of people and vehicles. Our results indicate these enhancements are likely to be effective in preventing FMD introduction and spread. Furthermore, these results will be useful to convince farmers of the need to comply with the strengthened control measures.

Considering the recent incidence of FMD in East Asian Countries [5,17,18], the possibility of reintroduction of FMD into Japan remains high. Therefore, continuous efforts, including further studies on FMD outbreaks, are crucial to improve containment and prevention measures against FMD.

## Conclusions

A case–control study was conducted to investigate risk factors associated with FMD transmission between farms during the 2010 Japan epidemic. In the northern part of the epidemic area, in the movement restriction area, the disease was likely to spread irrespective of the movement of people and vehicles, and physical barriers around the farms reduced disease transmission. In the southern part of the epidemic area, in the shipment restriction area, the disease seemed to be transmitted by the unrestricted movements of people and vehicles. These results provide insights for understanding the risk factors associated with FMD transmission and are useful to enhance preventive measures against FMD.

## Competing interests

The authors declare that they have no competing interests.

## Authors’ contributions

NM, SK, TN, YH and TT participated in the design of the study. NM, SK, TN and TK collected data from the farms. NM performed all data handling and analysis, and drafted the manuscript. SK and YH supported the data analysis performed by NM. TY, YH and TT helped to draft the manuscript. All authors read and approved the final manuscript.

## Supplementary Material

Additional file 1 Questionnaire used to investigate risk factors associated with transmission of FMD between farms during the epidemic in Japan in 2010. Click here for file
