# Supplementary material for: Risk factors for the transmission of foot-and-mouth disease during the 2010 outbreak in Japan: a case–control study
Source: BMC Vet Res. 2013 Jul 24;9:150. doi: 10.1186/1746-6148-9-150 (PMC3724691; doi:10.1186/1746-6148-9-150)
Supplement: Additional file 1 — Questionnaire used to investigate risk factors associated with transmission of FMD between farms during the epidemic in Japan in 2010. [file 1746-6148-9-150-S1.pdf]

Survey Questionnaire (case-control study)

This research is conducted by National Institute of Animal Health in concert with Miyazaki Prefecture as part of survey of the FMD Epidemiological Survey Team that was established by Ministry of Agriculture, Forestry and Fisheries. Personal informations collected through this research will not open to the public.

This research is conducted to promote improvement of containment and prevention measures against FMD. Please reply to investigators about the situation of your farm during the FMD epidemic in 2010.

Farm name	Farm owner name

Farm adress

Survey date	Investigator name
2011. .	

1. General farm information

Q1. What is feeding system on your farm?

breeding／fattening／farrow-to-finish／other（　　）

Q2. How many livestock did you raise on your farm?

suckler calf:（　　）, heifer:（　　）, breeding:（　　）, fattening:（　　）

Q3. Did you belong to some company group?

Yes／No

2. Farm location

Q4. Make a brief layout of farm facilities【for investigator】

(barn, gate, road, feed tanks, compost depot, house, office and so on.)

3. People movement

Q5. Did your farm staffs visit other livestock farms?

Yes／No

Q6. Did employed staff work on your farm?

Yes／No

Q7. Did anyone (except for farm staffs) visit inside your farm?

Yes／No

Q8. （Q7. If yes）Who visited inside your farm?

veterinarians／agricultural technicians／staffs of livestock related companies／ other livestock farmers／other（　　）

4. Vehicle movement

Q9. Did feed transport vehicles (including vehicles of your farm) visited inside your farm?

Date of visit	Name of company

Q10. Did bedding transport vehicles visited inside your farm?

Date of visit	Name of company

Q11. Did carcass transport vehicles visited inside your farm?

Date of visit	Name of company

5. Farm management

Q12. Were farm equipments (e.g. tractor, roll baler) shared with other farms?

Yes／No

Q13. (Q12. If yes)Were they used outside of your farm?

Yes／No

Q14. Was manure taken out from the farm?

Yes／No

Q15. (Q14. If yes)Where was manure taken out to?

own field／horticultural field／shared compost pile／other (　　)

Q16. (Q14. If yes)Plot the site on a map【for investigator】.

